# A Bioinspired Hierarchical Fast Transport Network Boosting Electrochemical Performance of 3D Printed Electrodes

**DOI:** 10.1002/advs.202204751

**Published:** 2022-10-26

**Authors:** Bo Zhao, Jiawen Wu, Zhiqiang Liang, Wenkai Liang, He Yang, Dan Li, Wei Qin, Meiwen Peng, Yinghui Sun, Lin Jiang

**Affiliations:** ^1^ Institute of Functional Nano and Soft Materials (FUNSOM) Jiangsu Key Laboratory for Carbon‐Based Functional Materials & Devices Soochow University Suzhou Jiangsu 215123 P. R. China; ^2^ College of Energy Soochow Institute for Energy and Materials Innovations Key Laboratory of Advanced Carbon Materials and Wearable Energy Technologies of Jiangsu Province Soochow University Suzhou Jiangsu 215006 P. R. China

**Keywords:** 3D printing, biomimetic materials, electrochemical electrodes, mass transport, water splitting

## Abstract

Current 3D printed electrodes suffer from insufficient multiscale transport speed, which limits the improvement of electrochemical performance of 3D printed electrodes. Herein, a bioinspired hierarchical fast transport network (HFTN) in a 3D printed reduced graphene oxide/carbon nanotube (3DP GC) electrode demonstrating superior electrochemical performance is constructed. Theoretical calculations reveal that the HFTN of the 3DP GC electrode increases the ion transport rate by more than 50 times and 36 times compared with those of the bulk GC electrode and traditional 3DP GC (T‐3DP GC) electrode, respectively. Compared with carbon paper, carbon cloth, bulk GC electrode, and T‐3DP GC electrode, the HFTN in 3DP GC electrode endows obvious advantages: i) efficient utilization of surface area for uniform catalysts dispersion during electrochemical deposition; ii) efficient utilization of catalysts enables the high mass activity of catalysts and low overpotential of electrode in electrocatalytic reaction. The cell of 3DP GC/Ni‐NiO||3DP GC/NiS_2_ demonstrates a low voltage of only 1.42 V to reach 10 mA cm^−2^ and good stability up to 20 h for water splitting in alkaline conditions, which is superior to commercialized Pt/C||RuO_2_. This work demonstrates great potential in developing high‐performance 3D printed electrodes for electrochemical energy conversion and storage.

## Graphical abstract

A 3D printed reduced graphene oxide/carbon nanotube (3DP GC) electrode with a bioinspired hierarchical fast transport network is reported, which demonstrates superior electrochemical performance. This is the first study to systematically investigate the hierarchical mass transport properties of 3D printed electrodes and reveal the promoting effect of multiscale fast mass transport on the electrochemical performance of 3D printed electrodes.

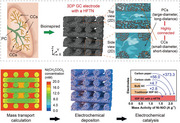

## 1 Introduction

Electrochemical energy conversion and storage technology promotes the rapid development of renewable energy and low‐carbon‐emission economy, representing a promising way to solve the increasingly serious energy shortage and climate issues. For the low‐cost and large‐scale application of electrochemical energy conversion and storage technology (such as batteries, supercapacitors, fuel cells, and water splitting), high electrochemical performance electrodes are urgently needed due to the great potential to improve the efficiency of electrical‐chemical conversion and reduce electrical energy consumption. Recently, due to the large surface area and high active material loading of 3D electrodes, which greatly contribute to better electrochemical performance compared to 2D planar electrodes, 3D electrodes have attracted extensive attention for use in electrochemical energy conversion and storage.^[^ ^1^, ^2^, ^3^, ^4^, ^5^, ^6^, ^7^ ^]^ For example, 3D electrodes such as carbon paper (CP),^[^ ^8^ ^]^ carbon cloth (CC),^[^ ^9^, ^10^ ^]^ 3D carbon fiber,^[^ ^11^, ^12^ ^]^ 3D graphene,^[^ ^13^, ^14^, ^15^ ^]^ Ni foam,^[^ ^16^, ^17^, ^18^ ^]^ and Cu foam^[^ ^19^ ^]^ demonstrate much better electrochemical performances than their 2D planar counterparts. Increasing the thickness of 3D electrodes can further improve the surface area and active material loading in electrochemical conversion and storage applications; however, the increased thickness brings longer transport distances of electrochemical reaction‐related species (such as ions and gases). Since the pores of conventional 3D electrodes are small and most of them are highly tortuous, it takes an excessively long time for the electrochemical reaction‐related species to be transported over long distances into the interior of 3D thick electrodes, which leads to a significant decrease in the utilization of surface area and active materials inside the electrode. Therefore, the transport limitation severely hinders the improvement of the electrochemical performance of 3D electrodes.

**Figure 1:**
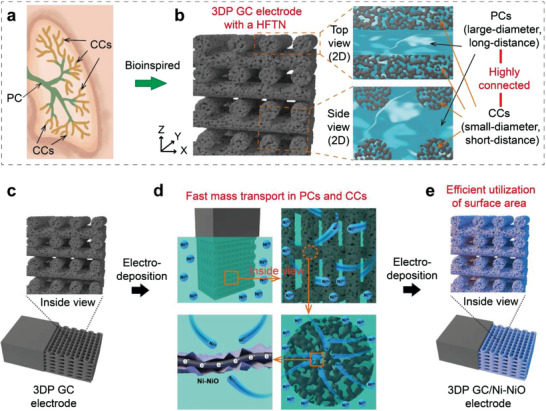
Design and electrochemical performance of a 3DP GC electrode with a bioinspired HFTN. a,b) Schematics of lung and 3DP GC electrodes with hierarchical transport networks. c–e) Schematics showing that PCs and CCs boost the surface area utilization of the 3DP GC electrode during electrochemical deposition.

Lung's hierarchical 3D transport network comprised of a highly connected parent channel (PC) and children channel (CC) can provide optimized transport efficiency and the lowest energy consumption and bring a large amount of available surface area (**Figure** **1**a). On the one hand, large‐diameter PCs can transport many gases from the outside air to CCs over long distances in a short time. On the other hand, a large number of small‐diameter CCs with high surface areas can transport gases from PCs to the surface of functional sites over short distances in a short period.^[^ ^20^, ^21^ ^]^ In addition, the high connectivity between PCs and CCs ensures unhindered and fast mass transfer, resulting in optimal transport efficiency at multiple scales. Recently, direct ink writing 3D printing technology has been widely used for rapidly forming various precursors, active or/and conductive materials by assembling the printed filaments into 3D printed electrodes with designed macroscopic grid channels.^[^ ^22^, ^23^, ^24^, ^25^, ^26^ ^]^ The macroscopic grid channels in 3D printed cubic lattice electrodes show fast transport performance similar to that of large‐diameter PCs, which can transport many reaction‐related species into the interior of several millimeter‐thick electrodes in a short time.^[^ ^27^, ^28^ ^]^ As a result, the electrochemical performance of 3D printed electrodes is greatly improved compared to traditional 3D electrodes in energy conversion and storage applications.^[^ ^29^, ^30^, ^31^, ^32^, ^33^ ^]^ However, most reported 3D printed electrodes always lack small‐diameter CCs connected to PCs, which hinders the optimization of transport efficiency and the improvement of active materials/surface area utilization of 3D printed electrodes, since the precise construction of the printed filament with microscopic open porous channels remains a big challenge. In addition, there is still a lack of systematic theoretical and experimental studies on the multiscale mass transport properties of 3D printed electrodes. Therefore, there is an urgent need to design and develop 3D printed electrodes with fast multiscale transport rates to greatly improve the electrochemical performance of 3D printed electrodes.

Herein, we construct a hierarchical fast transport network (HFTN) in a 3D printed reduced graphene oxide/carbon nanotube (3DP GC) electrode inspired by the lung's hierarchical transport network. The highly connected macroscopic grid channels and microscopic open porous channels in the HFTN are used as large‐diameter PCs and small‐diameter CCs, respectively, ensuring the sufficient utilization of the internal and outer surface areas of the 3DP GC electrodes (Figure 1b). The uniform dispersion of Ni‐NiO on the internal and outer surfaces after electrochemical deposition proves that the HFTN facilitates the highly efficient utilization of the internal and outer surface areas of 3DP GC electrodes (Figure 1c–e). Theoretical calculations further reveal that the printed macroscopic grid channels corresponding to PCs increase the ion transport rate by more than 50 times compared with the traditional bulk structure, and the microscopic open porous channels corresponding to CCs increase the ion transport rate by more than 36 times compared with the traditional microscopic closed porous structure. In the alkaline hydrogen evolution reaction (HER), HFTN enables the mass activity of Ni‐NiO on 3DP GC electrodes to be 373.3, 56.0, 4.7, and 2.8 times higher than those of conventional carbon paper (CP), carbon cloth (CC), bulk reduced graphene oxide/carbon nanotube (bulk GC), and traditional 3DP GC (T‐3DP GC) electrodes, respectively. The 3DP GC/Ni‐NiO electrode shows an overpotential of only 31.1 mV at 10 mA cm^−2^ and a Tafel slope of 35.7 mV dec^−1^, which is better than that of commercial Pt/C catalysts. After annealing and vulcanization, the NiS_2_ catalysts are also uniformly dispersed on the internal and outer surfaces of the 3DP GC electrode. In the alkaline oxygen evolution reaction (OER), the overpotential of the 3DP GC/NiS_2_ electrode is only 153 mV at 10 mA cm^−2^, which is better than that of commercial RuO_2_ catalysts. As the cathode and anode for water splitting, respectively, the 3DP GC/Ni‐NiO and 3DP GC/NiS_2_ electrodes show a voltage of only 1.42 V at 10 mA cm^−2^ and good stability up to 20 h. This work exhibits great potential in developing high‐performance 3D printed electrodes for electrochemical energy conversion and storage, such as batteries, supercapacitors, and electrocatalysis.

## 2 Results and Discussion

To fabricate 3D printed carbon‐based electrodes with good electrical conductivity, high mechanical strength, and tunable microstructure, we developed a series of partially reduced graphene oxide/carbon nanotube (prGO/CNT) inks. As shown in Figure S1 (Supporting Information), all the GO/CNT, prGO/CNT‐1, and prGO/CNT‐2 inks show high viscosity, good shear‐thinning properties, and adequate modulus. The scanning electron microscopy (SEM) characterizations demonstrate that the surface of the printed and freeze‐dried filament gradually changes from closed pores to open pores accompanied by the reduction of GO to prGO in the prGO/CNT ink (Figure S2a–c, Supporting Information). Raman spectroscopy further indicated that the D/G intensity ratio gradually increased and the oxygen functional groups of prGO gradually decreased during the reduction of GO to prGO (Figure S2d–f, Supporting Information). This may be because prGO/CNTs with fewer oxygen‐containing functional groups are easier to phase‐separate from water during the freeze‐drying process, resulting in an open porous microstructure prepared with ice crystals as a template. Using prGO/CNT‐2 ink with low oxygen‐containing functional groups, a cubic lattice 3DP GC electrode with good electrical conductivity and mechanical strength can be fabricated after rapid direct ink writing 3D printing, freeze‐drying, and high‐temperature annealing (Figures S3 and S4, Supporting Information).

As shown in Figure 1a,b, the 3DP GC electrode has a bioinspired HFTN. The 3D‐printed vertical macroscopic channels resemble the PCs of the lung, which are expected to transport a large amount of reaction‐related species in a short time during the electrochemical process. The microscopic open pores are similar to the CCs of the lung, which will exert the advantages of a short transmission distance and large available surface area during the electrochemical process. For example, the bioinspired HFTN will facilitate the simultaneous efficient utilization of the internal and outer surface areas of the 3DP GC electrode, enabling the uniform electrochemical deposition of Ni‐NiO catalysts on the internal and outer surfaces of the electrode (Figure 1c–e). PCs rapidly transport a large number of Ni^2+^ ions at the macroscopic scale, which is helpful for the electrochemical deposition of Ni‐NiO catalysts on the surface of electrode filaments; CCs further transport a large number of Ni^2+^ ions in the PCs to the interior of electrode filaments, effectively utilizing the internal surface of electrode filaments for electrochemical deposition of Ni‐NiO catalysts.

**Figure 2:**
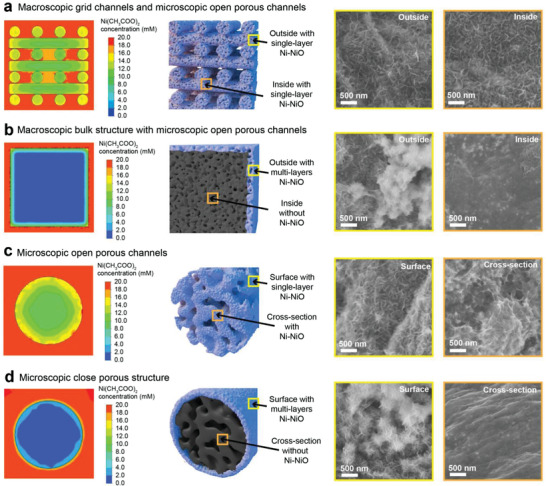
The influence of ions transport properties on the growth state of electrodeposited Ni‐NiO. The ions transport calculation, schematic diagram of the growth state of electrodeposited Ni‐NiO, and surface (cross‐section) scanning electron microscopy (SEM) characterization of a) macroscopic grid channels and microscopic open porous channels; b) macroscopic bulk structure with microscopic open porous channels; c) microscopic open porous channels; d) microscopic closed porous structure.

By employing a research method that combines fluid theoretical analysis (Figure S5, Supporting Information) of ion transport properties and electrochemical deposition experimental validation, we systematically investigate the hierarchical mass transport properties of the 3DP GC electrode and its advantages over conventional electrodes at the macro and microscales. First, the mass transport properties of the macroscopic PCs of the 3DP GC electrode and their advantages over conventional bulk electrodes are studied. Videos S1 and S2 (Supporting Information) demonstrate the fluid theoretical analysis of the macroscale ion transport process at the 3DP GC electrode and bulk GC electrode, respectively. As shown in **Figure** **2**a,b, the Ni^2+^ ion concentration in the PCs of the 3DP GC electrode reached the same concentration as that of the external electrolyte after 30 s of transfer, while almost no Ni^2+^ ions entered the bulk GC electrode. The distribution of Ni‐NiO on the electrode after electrochemical deposition is consistent with the theoretical calculation results. A layer of Ni‐NiO is uniformly grown on the internal and outer surfaces of the 3DP GC electrode, while Ni‐NiO is tightly packed only on the outer surface of the bulk electrode. Furthermore, by comparing the time to reach the same concentration in the same area outside the electrode (Figure S6, Supporting Information), we found that the PCs present an ion transport rate that was more than 50 times higher than that of the bulk structure. The vertically large‐diameter PC ensures the large‐scale high‐speed transport of Ni^2+^ ions, which is beneficial to the efficient electrochemical deposition reaction on the inner and outer surfaces of the 3DP GC electrodes.

Moreover, the mass transport properties of the microscopic CCs of the 3DP GC electrode and their advantages over conventional T‐3DP GC electrodes are studied. Videos S3 and S4 (Supporting Information) demonstrate the fluid theoretical analysis of the microscale ion transport process at the 3DP GC electrode and T‐3DP GC electrode, respectively. As shown in Figure 2c, the CCs of the 3DP GC electrode facilitate the rapid transport of Ni^2+^ ions into the interior of electrode filaments so that Ni‐NiO can be uniformly deposited on the surface and cross‐section of the electrode filaments. In contrast, the electrode filaments with traditional close porous structures display almost no ions entering, and Ni‐NiO is thickly deposited on the surface of electrode filaments (Figure 2d). In addition, CCs exhibited a more than 36‐fold higher ion transport rate than closed porous structures (Figure S7, Supporting Information). Therefore, PCs and CCs not only greatly enhance the ion transport rate of 3D printed electrodes but also provide a large amount of available surface area, which is proved by the BET measurment (Figure S8, Supporting Information).

**Figure 3:**
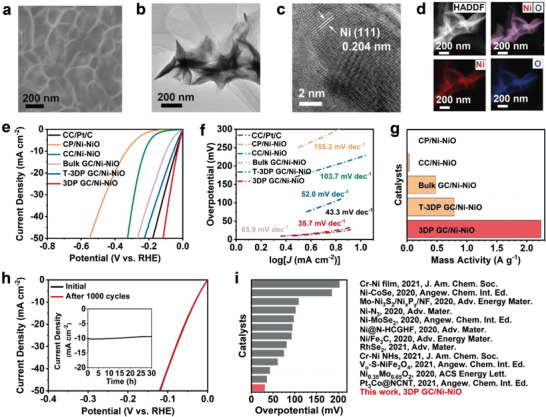
The morphology characterization and HER performance of 3DP GC/Ni‐NiO. a) SEM image of Ni‐NiO on a 3DP GC electrode. b) TEM image of Ni‐NiO nanosheets. c) HRTEM image of Ni‐NiO nanosheets. d) HAADF‐STEM images of Ni‐NiO nanosheets. e) HER polarization curves of Pt/C, CP/Ni‐NiO, CC/Ni‐NiO, Bulk GC/Ni‐NiO, T‐3DP GC/Ni‐NiO, 3DP GC/Ni‐NiO. f) Corresponding Tafel plots. g) Corresponding mass activities. h) LSV curves before and after 1000 CV cycles of 3DP GC/Ni‐NiO (Inset: long‐term stability test operated at overpotential of 36 mV.) i) Overpotentials are required at 10 mA cm^−2^ among 3DP GC/Ni‐NiO and the reported HER catalysts in 1.0 m KOH.^[^ ^34^, ^35^, ^36^, ^37^, ^38^, ^39^, ^40^, ^41^, ^42^, ^43^, ^44^ ^]^

The composition and morphology of Ni‐NiO on 3DP GC were investigated by SEM and transmission electron microscopy (TEM), as shown in **Figure** **3**a–d. A nanosheet array structure of Ni‐NiO was grown on the 3DP GC surface (Figure 3a,b). As depicted in Figure S9 (Supporting Information), the X‐ray diffraction (XRD) pattern shows diffraction peaks of 3DP GC/Ni‐NiO consistent with those of Ni (JCPDS Card no. 04–0850). A high‐resolution TEM (HRTEM) image displays crystalline Ni with lattice fringes spaced at 0.204 nm (Figure 3c), corresponding to the (111) crystal plane of Ni, which is consistent with XRD characterization. High‐angle annular dark‐field scanning TEM (HAADF‐STEM) and elemental mapping display the homogenous distribution of nickel and oxide elements over the Ni‐NiO nanosheet array (Figure 3d). In addition, X‐ray photoelectron spectroscopy (XPS, Figure S10, Supporting Information) spectra further proved the existence of metallic Ni and Ni oxides in Ni‐NiO because of the two peaks at 852.3 and 869.7 eV. In addition, the existence of NiO was proved by 855.3 and 873.4 eV, which represent the characteristic Ni 2p3/2 and 2p1/2 of Ni^2+^. The remaining two peaks at 861.3 and 881.0 eV were the satellite peaks. Compared with that of NiO, the peaks with binding energies of 856.5 and 874.3 eV were indexed to Ni^2+^ signals, while the peaks at 861.8 and 880.7 eV represent two satellite peaks of Ni.

To demonstrate the advantages of the 3DP GC electrode in electrochemical catalytic performance, the hydrogen evolution reaction (HER) performance of different electrodes was investigated in 1.0 m KOH with the same Ni‐NiO loading of 17.9 mg cm^−2^. As shown in Figure 3e, 3DP GC/Ni‐NiO can achieve a current density of 10 mA cm^−2^ at a low overpotential of only 31.1 mV, which is superior to those of T‐3DP GC/Ni‐NiO, bulk GC/Ni‐NiO, CP/Ni‐NiO, CC/Ni‐NiO, and 20% Pt/C. In addition, the Tafel slope of 3DP GC/Ni‐NiO is 35.7 mV dec^−1^, which is also much lower than those of T‐3DP GC/Ni‐NiO (52.0 mV dec^−1^), bulk GC/Ni‐NiO (65.9 mV dec^−1^), CP/Ni‐NiO (155.2 mV dec^−1^), CC/Ni‐NiO (103.7 mV dec^−1^), and 20% Pt/C (43.3 mV dec^−1^) (Figure 3f). The mass activity of Ni‐NiO on the 3DP GC electrode is 2.24 A g^−1^ at an overpotential of 100 mV, which is 2.8, 4.7, 56.0, and 373.3 times greater than that of Ni‐NiO on T‐3DP GC (0.79 A g^−1^), bulk GC (0.48 A g^−1^), CC (0.04 A g^−1^), and CP (0.006 A g^−1^) electrodes (Figure 3g), respectively. Compared with the bulk GC/Ni‐NiO electrode, the macroscopic grid transport channel of the 3DP GC/Ni‐NiO electrode promotes the utilization of the internal surface area of the electrode and possesses more active sites in the HER (Figure S11, Supporting Information). In addition, compared with the T‐3DP GC/Ni‐NiO, T‐3DP GC‐1/Ni‐NiO, and T‐3DP GC‐2/Ni‐NiO electrodes, the microscopic open porous structure of the 3DP GC/Ni‐NiO electrodes is beneficial for utilizing the surface and cross‐section of filaments simultaneously, providing more active sites in the HER. Therefore, under the same Ni‐NiO loading, 3DP GC/Ni‐NiO with a bioinspired hierarchical transport network presents better electrochemical catalytic performance than conventional 3D electrodes and 3D printed electrodes. As shown in Figure 3h, the 3DP GC/Ni‐NiO displays excellent stability after 30 h of LSV polarization and 1000 CV cycles. Moreover, 3DP GC/Ni‐NiO can achieve a current density of 10 mA cm^−2^ with a low overpotential of 31.1 mV, which is also superior to most of the reported nonnoble metal‐based electrocatalysts (Figure 3i). In addition, the HER performance of 3DP GC/Ni‐NiO outperforms all reported single‐metal Ni‐based catalysts and is especially significantly better than the reported electrocatalytic electrodes based on Ni‐NiO catalysts (Figure S12, Supporting Information).

The inherent nature of 3DP GC/Ni‐NiO with HFTN exhibiting better HER performance than T‐3DP GC/Ni‐NiO, bulk GC/Ni‐NiO, CP/Ni‐NiO, and CC/Ni‐NiO was investigated using SEM and electrocatalysis. As shown in the SEM images of Figure 2; and Figure S13 (Supporting Information), Ni‐NiO is uniformly dispersed on both the inner and outer surfaces of the 3DP GC electrode; nevertheless, Ni‐NiO is tightly packed on the surface of the T‐3DP GC, bulk GC, CP, and CC electrodes. The electrochemical active surface area (ECSA) test shows that the double‐layer capacitance (*C*_dl_) of 3DP GC/Ni‐NiO is 1.54 mF cm^−2^, which is significantly better than those of T‐3DP GC/Ni‐NiO (0.43 mF cm^−2^), bulk GC/Ni‐NiO (0.23 mF cm^−2^), CC/Ni‐NiO (0.16 mF cm^−2^), and CP/Ni‐NiO (0.03 mF cm^−2^) (Figures S14 and S15, Supporting Information). The above results indicate that HFTN not only promotes the uniform dispersion of highly loaded Ni‐NiO but also ensures the efficient utilization of active sites on Ni‐NiO. The possible reasons are illustrated in Figure S16 (Supporting Information): during the HER process, the ion and electron transport rates on the uniformly dispersed Ni‐NiO are very fast, so that the abundant active sites of Ni‐NiO are effectively utilized and enable Ni‐NiO to exhibit high mass activity; in contrast, the surfaces of Ni‐NiO on the T‐3DP GC, bulk GC, CP, and CC electrodes were partially covered by the aggregated Ni‐NiO due to insufficient electron and ion transport, resulting in fewer active sites and low mass activity of Ni‐NiO. Therefore, under the same Ni‐NiO loading, the 3DP GC/Ni‐NiO with the higher mass activity of Ni‐NiO demonstrates better HER catalytic performance than those of T‐3DP GC/Ni‐NiO, bulk GC/Ni‐NiO, CP/Ni‐NiO, and CC/Ni‐NiO.

**Figure 4:**
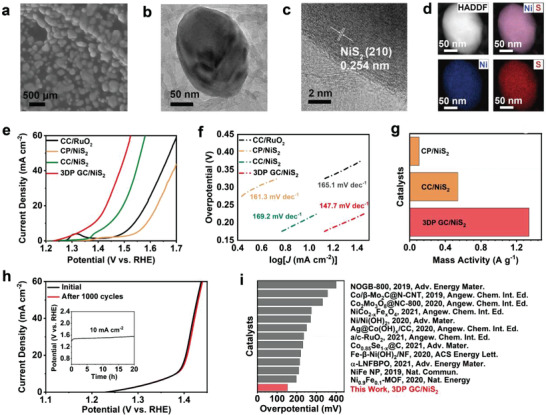
The morphology characterization and OER performance of 3DP GC/NiS_2_. a) SEM image of NiS_2_ nanoparticles on a 3DP GC electrode. b) TEM image of NiS_2_ nanoparticles. c) HRTEM image of NiS_2_ nanoparticles. d) HAADF‐STEM images of NiS_2_ nanoparticles. e) OER polarization curves, f) corresponding Tafel plots, and g) the mass activity for CP/NiS_2_, CC/NiS_2_, and 3DP GC/NiS_2_. h) LSV curves before and after 1000 CV cycles. (Inset: long‐term stability test operated at an overpotential of 151 mV) i) Overpotentials required at 10 mA cm^−2^ among 3DP GC/NiS_2_ and the reported OER catalysts in 1.0 m KOH.^[^ ^45^, ^46^, ^47^, ^48^, ^49^, ^50^, ^51^, ^52^, ^53^, ^54^, ^55^, ^56^ ^]^

The 3DP GC/Ni‐NiO electrode was vulcanized for efficient application in the oxygen evolution reaction (OER). After vulcanization treatment (Figures S17 and S18, Supporting Information), the structure of the 3DP GC electrode remains unchanged and Ni‐NiO on 3DP GC was transformed into NiS_2_ nanoparticles (**Figure** **4**a,b). The XRD pattern shows diffraction peaks of 3DP GC/NiS_2_ consistent with those of Ni (JCPDS No. 88‐1709) (Figure S19, Supporting Information). The HRTEM image displays crystalline NiS_2_ with a lattice fringe spaced at 0.254 nm, corresponding to the (210) crystal plane of NiS_2_, which is consistent with the XRD characterization (Figure 4c). HAADF‐STEM and elemental mapping display the homogenous distribution of nickel and sulfur over the NiS_2_ nanoparticles (Figure 4d). In addition, XPS was employed to investigate the chemical composition and valence state of 3DP GC/NiS_2_ (Figure S20, Supporting Information). In the high‐resolution Ni 2p spectra, the peaks at 853.2 and 870.8 eV represent the characteristic Ni 2p_3/2_ and 2p_1/2_ of Ni^2+^, respectively. And the corresponding satellite peaks centered at 857.7 and 876.5 eV. The presence of S 2p_1/2_ (163.9 eV) and S 2p_3/2_ (162.8 eV) peaks in the high‐resolution S 2p spectra confirms the Ni‐S bonds in NiS_2_. In addition, the SEM images of NiS_2_ on different electrodes are shown in Figure S21 (Supporting Information). As shown in Figure 4e, 3DP GC/NiS_2_ can achieve a current density of 10 mA cm^−2^ with only a low overpotential of 153 mV, which is superior to CC/NiS_2_ (214 mV), CP/NiS_2_ (356 mV), and RuO_2_ (308 mV). In addition, the Tafel slope of 3DP GC/NiS_2_ is 147.7 mV dec^−1^, which is also better than that of CC/NiS_2_ (169.2 mV dec^−1^), CP/NiS_2_ (161.3 mV dec^−1^), and RuO_2_ (165.1 mV dec^−1^) (Figure 4f; and Figure S18, Supporting Information). The outstanding electrochemical catalytic performance of 3DP GC/NiS_2_ is due to the mass activity of NiS_2_ on the 3DP GC electrode being 2.5 and 12.7 times that of NiS_2_ grown on CC and CP electrodes, respectively (Figure 4g). As shown in Figure 4h, the 3DP GC/NiS_2_ displays excellent stability during 20 h of LSV polarization and 1000 CV cycles. Moreover, the overpotential of 3DP GC/NiS_2_ of 153 mV at a current density of 10 mA cm^−2^ is superior to that of most of the reported nonnoble metal‐based electrocatalysts (Figure 4i).

In addition, we performed detailed characterization of the catalyst after the OER reaction. As shown in Figure S22a (Supporting Information), Raman spectroscopy confirmed the formation of NiOOH after the OER reaction. The XRD pattern shows that the peak intensity of NiS_2_ is significantly weakened after the OER reaction (Figure S22b, Supporting Information), implying a decrease in the proportion of NiS_2_ in the catalyst. Figure S22c,d (Supporting Information) demonstrates the coexistence of NiS_2_ and NiOOH in the catalyst after the OER reaction. The HAADF‐STEM images confirmed that the proportion of sulfur in the catalyst was significantly reduced after the OER reaction, while the proportion of oxygen was significantly increased (Figure S22e, Supporting Information). In addition, the OER performance of 3DP GC/NiS_2_‐NiOOH is still much better than that of CC/NiS_2_‐NiOOH and CP/NiS_2_‐NiOOH despite the partial conversion of NiS_2_ to NiOOH in the OER (Figure S23, Supporting Information). The above results fully demonstrate that our 3DP GC electrode exhibits better electrochemical performance than traditional CC and CP electrodes in different catalyst systems.

**Figure 5:**
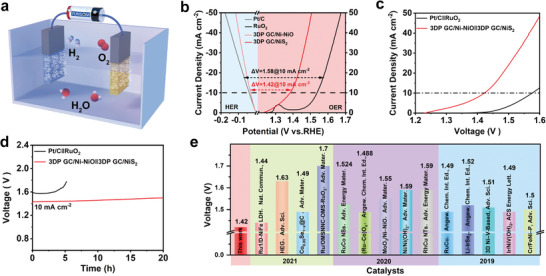
The overall water splitting performance of 3DP GC/Ni‐NiO and 3DP GC/NiS_2_. a) Schematic diagram of overall water splitting. b) Polarization curves for the HER and OER. c) LSV curves of the 3DP GC/Ni‐NiO||3DP GC/NiS_2_ and Pt/C||RuO_2_ for water electrolysis in 1.0 m KOH at a scan rate of 5 mV s^−1^. d) Long‐term stability test for overall water splitting of 3DP GC/Ni‐NiO||3DP GC/NiS_2_ and Pt/C||RuO_2_ at 10 mA cm^−2^. e) Voltage required at 10 mA cm^−2^ among 3DP GC/Ni‐NiO||3DP GC/NiS_2_ and the reported catalysts for water electrolysis in 1.0 m KOH ^[^ ^49^, ^54^, ^57^, ^58^, ^59^, ^60^, ^61^, ^62^, ^63^, ^64^, ^65^, ^66^, ^67^, ^68^ ^]^

Since the 3DP GC/Ni‐NiO and 3DP GC/NiS_2_ electrodes demonstrate efficient HER and OER activity in 1.0 m KOH electrolyte, we further employed them as the cathode and anode for a two‐electrode water splitting cell in alkaline (**Figure** **5**a). The cell of 3DP GC/Ni‐NiO||3DP GC/NiS_2_ requires a voltage as low as 1.42 V to achieve a current density of 10 mA cm^−2^, which is superior to that of commercialized CC/Pt/C||CC/RuO_2_ (1.58 V) (Figure 5b,c; and Figure S24, Supporting Information). In addition, compared with the fast decay of the catalytic performance of Pt/C||RuO_2_, 3DP GC/Ni‐NiO||3DP GC/NiS_2_ presents very good stability in the long‐term water splitting of 20 h (Figure 5d). Moreover, the voltage of 1.42 V at a current density of 10 mA cm^−2^ of 3DP GC/Ni‐NiO||3DP GC/NiS_2_ is superior to most of the recently reported nonnoble metal‐based electrocatalysts (Figure 5e).

## 3 Conclusion

Inspired by the lung's hierarchical transport network, we have constructed a 3DP GC electrode with HFTN via 3D printing and freeze casting. The macroscopic grid channels and microscopic open porous channels in the HFTN are used as PCs and CCs, respectively. Theoretical calculations reveal that 3DP GC electrodes with HFTN exhibit much higher ion transport rates than conventional T‐3DP GC electrodes with PCs and bulk GC electrodes with CCs. Electrochemical deposition experiments demonstrate that HFTN ensures the efficient utilization of the inner and outer surface areas of the 3DP GC electrode for the uniform growth of a Ni‐NiO layer accompanied by abundant active sites. The HER and OER tests proved that HFNT can guarantee the high mass activity of the catalysts with high loading, presenting superior electrocatalytic performances of 3DP GC/Ni‐NiO and 3DP GC/NiS_2_ in alkaline conditions, respectively. In addition, the cell of 3DP GC/Ni‐NiO||3DP GC/NiS_2_ shows a voltage of only 1.42 V at 10 mA cm^−2^ and good stability up to 20 h for water splitting, which is superior to commercialized Pt/C||RuO_2_. This work paves the way for the development of 3D printed electrodes with fast transport properties and high electrochemical performance.

## 4 Experimental Section

### Materials

Graphite flakes (XF049, 50 mesh) were purchased from XFNANO Inc. Multiwalled carbon nanotubes (CNTS‐010‐0) were purchased from Tanfeng Tech. Inc. Concentrated H_2_SO_4_ (98%), fuming nitric acid (99%), and hydrochloric acid (36%) were purchased from Chinasun Specialty Products. KMnO_4_ (99.5%), P_2_O_5_ (98%), H_2_O_2_ (30%) solution, and K_2_S_2_O_8_ (99.5%) were purchased from Sinopharm Chemical Reagent. L‐Ascorbic acid (L‐AA) (99.99%) was purchased from Sigma‐Aldrich. Ni(CH_3_COO)_2_∙6H_2_O (99.9%), RuO_2_ (99.9%), and Pt/C (20 wt %) were purchased from Macklin. Nafion (5%) ethanol solution and carbon black were obtained from Alfa Aesar. Potassium hydroxide (99.99%) and thiourea (99.999%) were obtained from Sinopharm Chemical Reagent Co., Ltd. All the materials were used as received.

### Synthesis of Partially Reduced GO (prGO) Ink

GO was synthesized with a modified Hummers method. L‐AA (1 mg mL^−1^) and GO (1 mg mL^−1^) were mixed in deionized water (DI) with vigorous stirring. Then, the mixture was heated to 65 °C and kept for 45 min, and the unreacted L‐AA was washed away by centrifugation. After being concentrated by suction filtration, the pr‐GO inks were prepared.

### Synthesis of Acidified CNT Ink

CNT (1 g), concentrated nitric acid (22 mL), and concentrated sulfuric acid were added to a single‐necked flask and reacted at 90 °C for 90 min to obtain acidified CNT. The CNTs were washed with DI water several times and then filtered to obtain concentrated CNT ink.

### Synthesis of Printing Ink

pr‐GO ink and acidified CNT ink were added into a beaker with the addition of DI water, and the solution was stirred for 6 h.

### Synthesis of 3DP GC, T‐3DP GC, and Bulk GC

The 3DP GC electrode was printed by an extrusion‐based 3D printing method. The homemade 3D printer is modified from an industrial 3D robotic dispenser system (Fisnar F5200N benchtop gantry, Wayne, NJ) with the capability for programmable patterning in three dimensions. A high‐precision pressure controller was used to provide controllable printing pressure to extrude ink filaments smoothly. In the experiments, the nozzle diameter, pressure, and nozzle's moving speed were 400 µm, 180–360 kPa, and 5–30 mm s^−1^, respectively. The target patterns were printed onto glass wafers in air at room temperature. It takes approximately 160 s to print 18 layers of 3DP GC electrodes with a size of 1 × 2 cm^−2^. After printing, the printed architectures were frozen in a −80 °C refrigerator for 0.5 h and lyophilized for 12 h. Then, the samples were thermally reduced at 900 °C in N_2_ for 120 min with a heating/cooling rate of 5 °C min^−1^ to obtain 3DP GC electrodes. Under the same conditions as the preparation of 3DP GC, T‐3DP GC with a nonporous microscopic surface was fabricated by controlling the GO reduction time, and bulk GC with a porous microscopic surface was prepared by adjusting the 3D printing program.

### Synthesis of 3DP GC/Ni‐NiO, T‐3DP GC/Ni‐NiO, Bulk GC/Ni‐NiO, CC/Ni‐NiO, and CP/Ni‐NiO

3DP GC electrodes were first pretreated by O_2_ plasma for 3 min. Then, Ni‐NiO was grown on 3DP GC (T‐3DP GC, Bulk GC/Ni‐NiO, CC/Ni‐NiO, CP/Ni‐NiO) by a facile electrodeposition process in a 20 mm Ni(CH_3_COO)_2_ aqueous electrolyte. The chronoamperometry method with −3 V versus SCE for 1 h was used in this process. The obtained sample was washed with DI water three times and dried at 70 °C.

### Synthesis of 3DP GC/NiS_2_, CC/NiS_2,_ and CP/NiS_2_

The 3DP GC/Ni‐NiO, CC/Ni‐NiO, and CP/Ni‐NiO electrodes were placed on one side of a tube furnace (upstream of gas flow), thiourea was placed on the other side (downstream), and the tube furnace was calcined at 500 °C for 60 min under an Ar atmosphere. Then, the 3DP GC/NiS_2_, CC/NiS_2,_ and CP/NiS_2_ electrodes were obtained.

### Characterizations

Microscopic morphology characterization was measured by SEM (Carl Zeiss Supra 55) operated at 10 kV. HRTEM images, HAADF‐STEM images, and STEM‐EDS mapping images were obtained by an FEI Tecnai F20 operated at 200 kV. X‐ray diffraction (XRD) was obtained by a PANalytical B.V. Empyrean X‐ray diffractometer with Cu‐K*α* radiation. X‐ray photoelectron spectroscopy (XPS) was performed on an ESCALAB 250 XI XPS spectrometer.

### Electrochemical Measurements

All measurements of electrochemical performance were investigated on an electrochemical workstation (CHI 660E) in a three‐electrode system at room temperature, in which the obtained electrodes were employed as the working electrode, graphite rods (HERs), Pt plates (OERs) acted as counter electrodes and saturated calomel electrodes served as reference electrodes. The linear sweep voltammetry (LSV) polarization curve was obtained with a scan rate of 5 mV s^−1^. All the obtained data were corrected by 100% iR compensation, and the potentials were converted to reversible hydrogen electrode (RHE), *E*_RHE_=*E*_SCE_+0.059pH+0.242. Electrochemical impedance spectroscopy (EIS) was obtained in a KOH electrolyte with a frequency range of 0.01–10^5^ Hz. The ECSA was collected by CV at scan rates ranging from 1 to 5 mV s^−1^. The overall water splitting performance was studied in a two‐electrode system. The 3DP GC/Ni‐NiO electrode worked as the cathode, and the 3DP GC/NiS_2_ acted as the anode. The stability of the electrode was tested under 10 mA cm^−2^ at room temperature.

## Conflict of Interest

The authors declare no conflict of interest.

## Author Contributions

B.Z., J.W.W., and Z.Q.L. contributed equally to this work. L.J., Y.H.S.. and M.W.P. conceived the idea and supervised the project. B.Z., J.W.W., and Z.Q.L. carried out the experiments. B.Z., M.W.P., J.W.W., Z.Q.L., W.Q., W.K.L., H.Y., and D.L. contributed to the characterizations and electrochemical tests. M.W.P. conducted the fluid theoretical calculations. M.W.P., B.Z., J.W.W., Z.Q.L., and Y.H.S. analyzed the data and wrote the manuscript. All authors discussed and commented on the manuscript.

## Supporting information

Supporting Information

Supplemental Video 1

Supplemental Video 2

Supplemental Video 3

Supplemental Video 4

## Data Availability

The data that support the findings of this study are available from the corresponding author upon reasonable request.
