# Supplementary material for: A Bioinspired Hierarchical Fast Transport Network Boosting Electrochemical Performance of 3D Printed Electrodes
Source: Adv Sci (Weinh). 2022 Oct 26;9(35):2204751. doi: 10.1002/advs.202204751 (PMC9762319; doi:10.1002/advs.202204751)
Supplement: Supplementary file 1 — Supporting Information [file ADVS-9-2204751-s001.pdf]

ADVANCED SCIENCE
Open Access

Supporting Information

for *Adv. Sci.*, DOI 10.1002/advs.202204751

A Bioinspired Hierarchical Fast Transport Network Boosting Electrochemical Performance of 3D Printed Electrodes

Bo Zhao, Jiawen Wu, Zhiqiang Liang, Wenkai Liang, He Yang, Dan Li, Wei Qin, Meiwen Peng, Yinghui Sun* and Lin Jiang**

Supporting Information

A Bioinspired Hierarchical Fast Transport Network Boosting Electrochemical Performance of 3D Printed Electrodes

Bo Zhao[1,⊥], *Jiawen Wu*[1,⊥], *Zhiqiang Liang*[1,⊥], *Wenkai Liang*[1], *He Yang*[1], *Dan Li*[1], *Wei Qin*[1], *Meiwen Peng*[1,*], *Yinghui Sun*[2,*], *and Lin Jiang*[1,*]

1. Institute of Functional Nano and Soft Materials (FUNSOM), Jiangsu Key Laboratory for Carbon-Based Functional Materials & Devices, Soochow University, Suzhou 215123, Jiangsu, P. R. China
2. College of Energy, Soochow Institute for Energy and Materials Innovations, Key Laboratory of Advanced Carbon Materials and Wearable Energy Technologies of Jiangsu Province, Soochow University, Suzhou 215006, Jiangsu, P. R. China

⊥ These authors contributed equally to this work

Corresponding author E-mail: mwpeng@suda.edu.cn (M. W. Peng); yinghuisun@suda.edu.cn (Y. H. Sun); ljiang@suda.edu.cn (L. Jiang)

Mass transfer simulation:

(1) Calculation model

A cylindrical material with a diameter of 510 μm and a height of 510 μm, a grid scaffold and a bulk with a size of 3500*3500 μm^2 are put into a container with 20 mM $Ni(CH_3COO)_2$.

(2) Meshing

The entire computational domain is meshed through a polyhedral mesh and is divided into two parts: the porous material part and the container part except for the porous material. Cylindrical material: The total number of grids is 119114, of which the number of grids in the porous material part is 30249 and the number of grids in the container part is 88865. Grid scaffold and bulk: The total number of grids is 254213, of which the number of grids in the porous material part is 103215, and the number of grids in the container part is 150998.

(3) Boundary conditions

The upper surface of the container is the free pressure outlet boundary, and the outlet pressure is 0 Pa; the other 5 surfaces are all smooth and nonslip wall boundaries. The interface between the porous area and the container is the interior boundary. Define the porous material domain: set the porous area with the parameters shown in the figure below, where fluid porosity is set to 0.5 for materials with holes on the side and 1 for materials with no holes on the side.

(4) Calculation model settings

The species transport component transport model is opened in FLUENT and defines the nickel acetate solution mixture. The two components are water and nickel acetate. The standard K-e model is used as the turbulence model. The concentration of nickel acetate in the outer container is defined as 20 mM, and the concentration of nickel acetate in the porous material area is defined as 0 in the initial state.

(5) Calculation results

In the mass transfer animation, a certain section is selected as the object of investigation.

Supplementary Figures

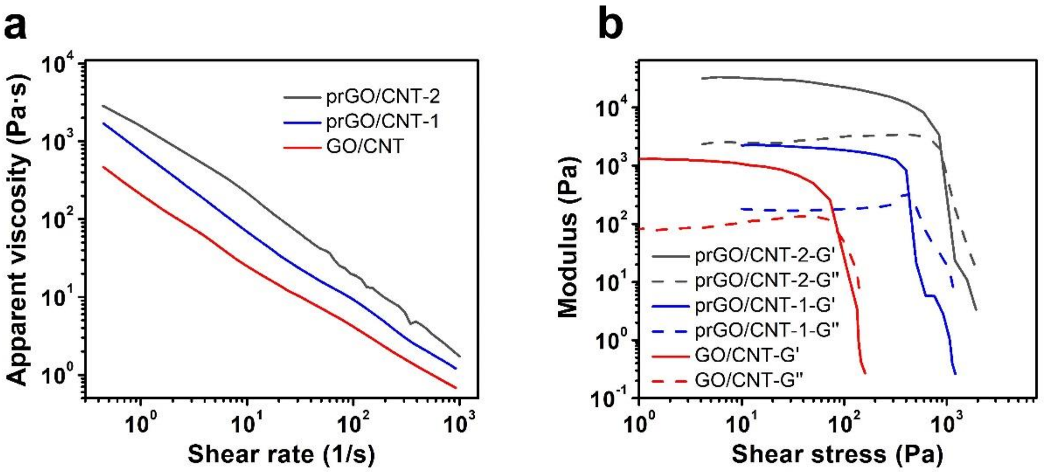

Figure S1. (a) Apparent viscosity as a function of shear rate for GO/CNT and pr-GO/CNT inks. (b) Storage modulus (G') and loss modulus (G'') as a function of shear stress for GO/CNT and pr-GO/CNT inks.

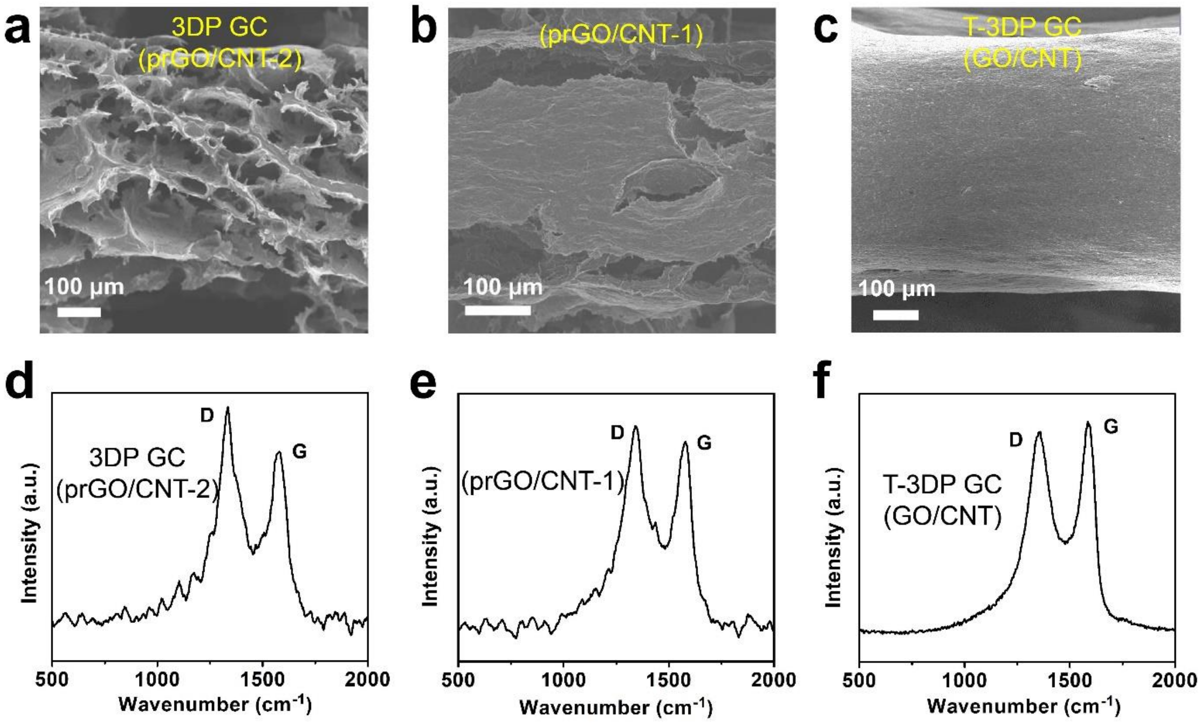

Figure S2. (a-c) SEM images and (d-f) Raman spectra of 3DP GC with tunable microstructure.

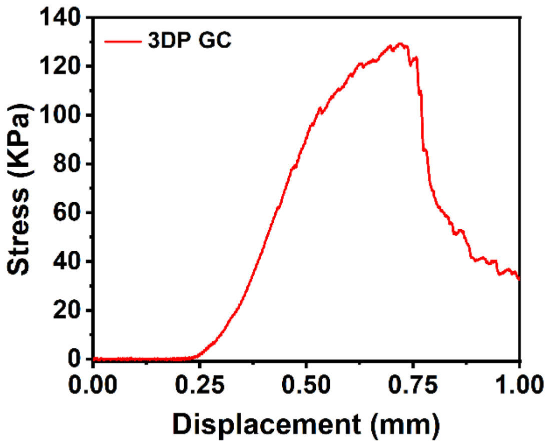

Figure S3. Flexural stress as a function of roller displacement (blending deflection) for 3DP GC electrode.

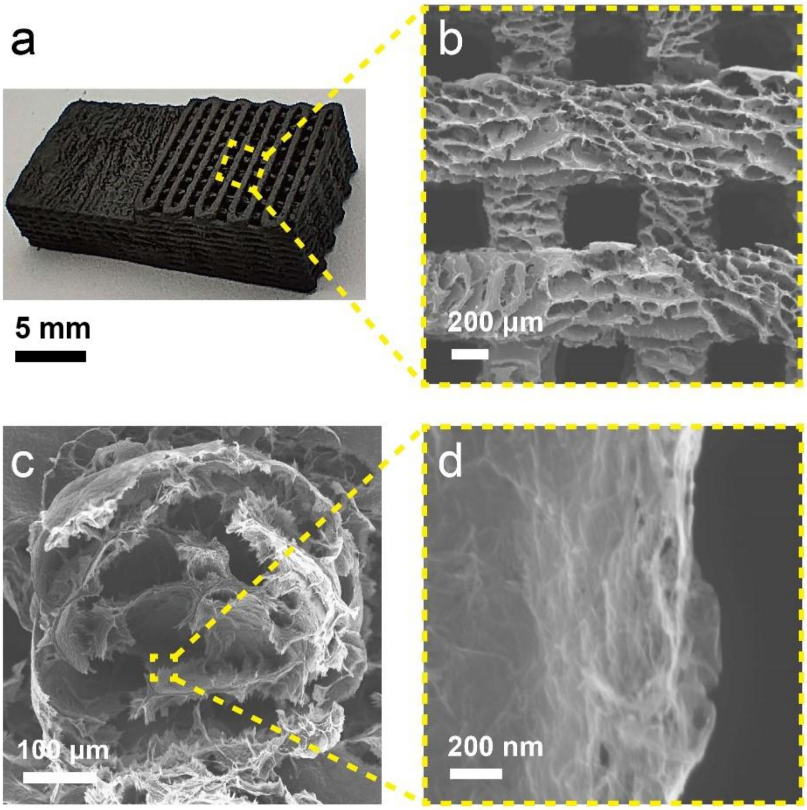

Figure S4. Optical and SEM images of 3DP GC electrode.

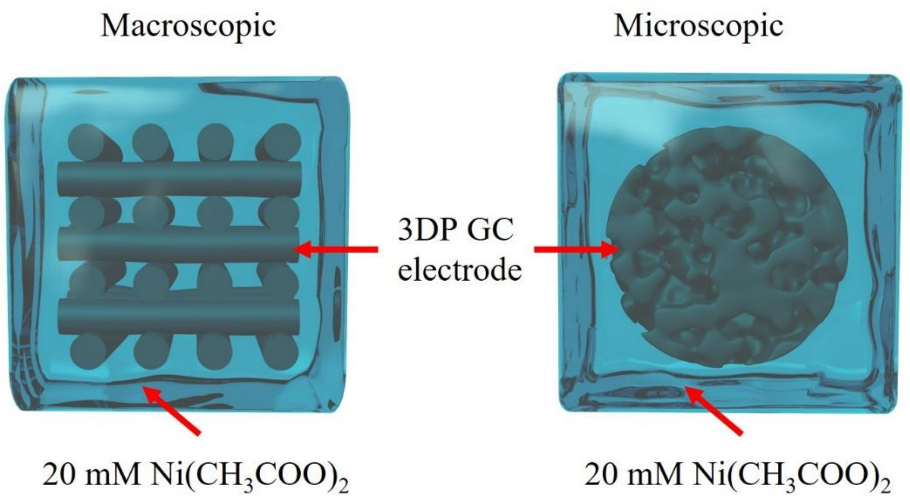

Figure S5. Salt transportation model of 3DP GC electrodes.

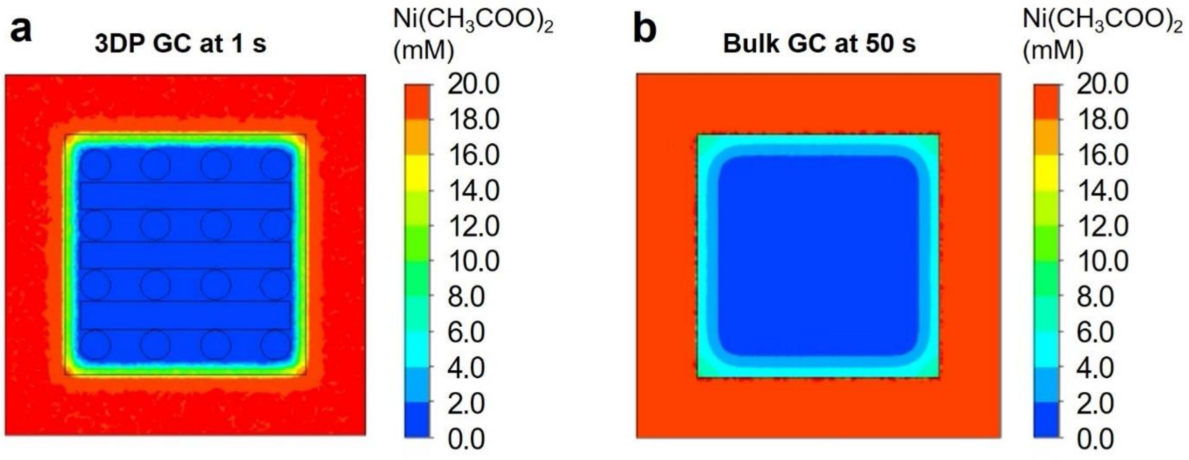

Figure S6. Simulation of $Ni(CH_3COO)_2$ transfer under different 3D electrodes. (a) 3DP GC. (b) Bulk GC.

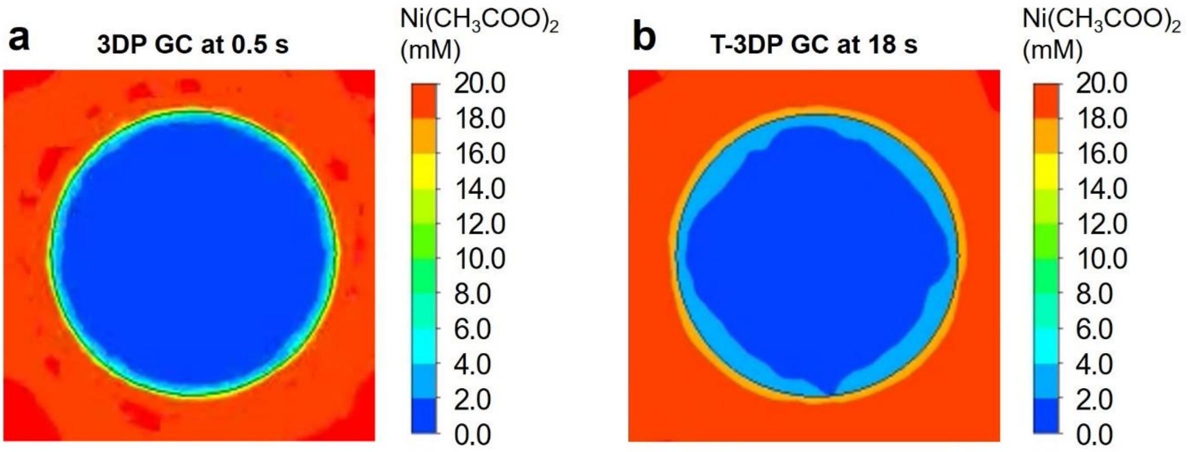

Figure S7. Simulation of $Ni(CH_3COO)_2$ transfer under different 3D electrodes. (a) 3DP GC. (b) T-3DP GC.

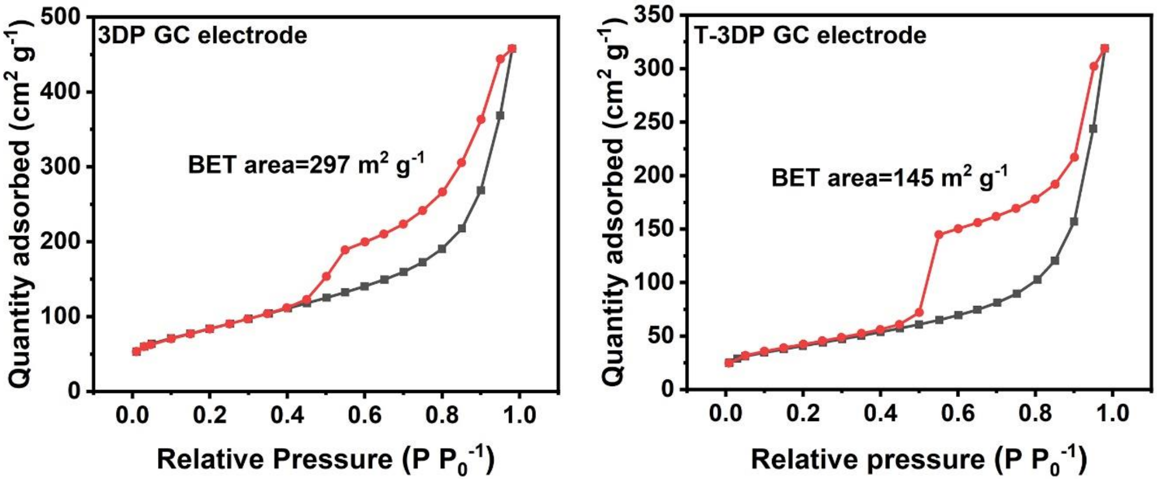

Figure S8. Nitrogen adsorption/desorption isotherm of 3DP GC electrode and T-3DP GC electrode.

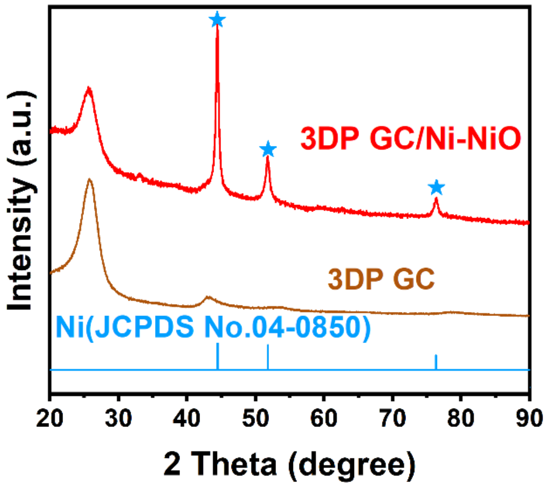

Figure S9. XRD patterns of 3DP GC Ni-NiO electrode.

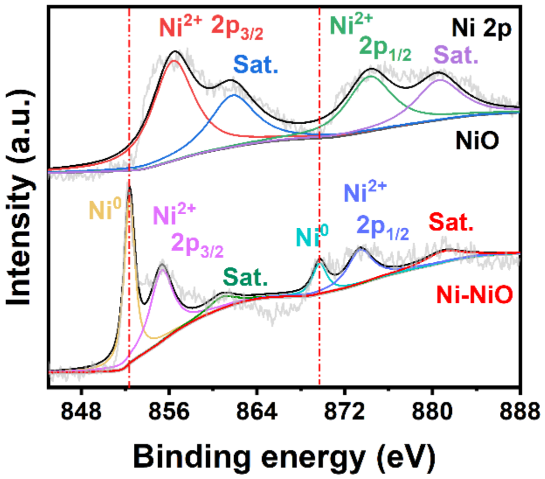

Figure S10. XPS spectra of NiO and Ni-NiO on 3DP GC electrode.

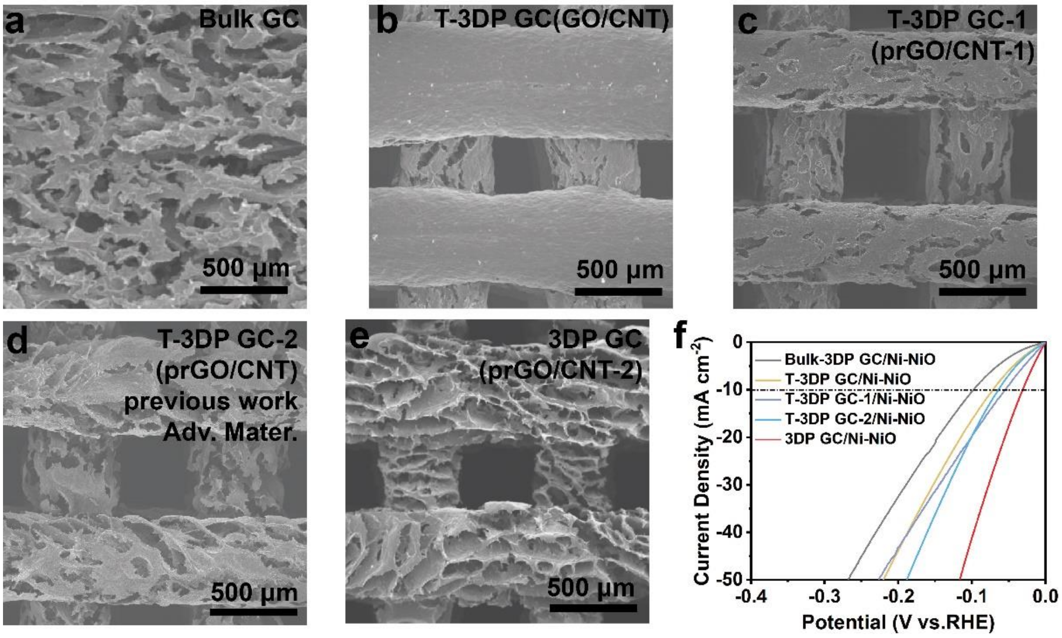

Figure S11. SEM images of (a) bulk GC electrode, (b-d) traditional 3DP GC electrodes and (e) 3DP GC electrode with HFTN. (f) HER polarization curves of bulk GC/Ni-NiO, T-3DP GC/Ni-NiO, T-3DP GC-1/Ni-NiO, T-3DP GC-2/Ni-NiO and 3DP GC/Ni-NiO.

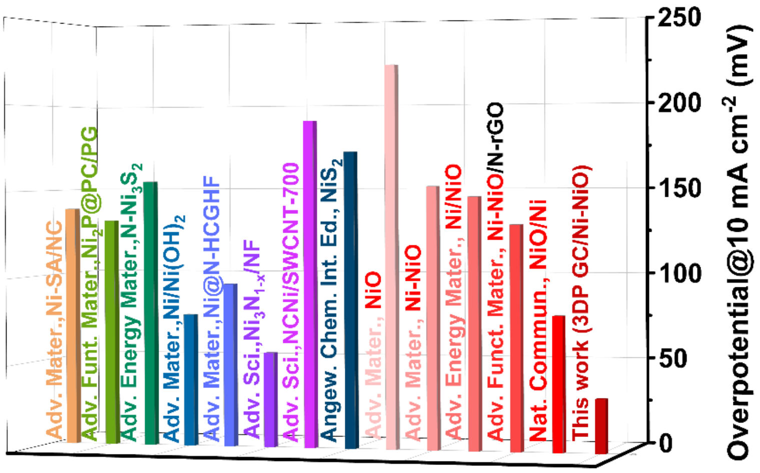

Figure S12. Overpotentials are required at 10 mA • cm^{-2} among 3DP GC/Ni-NiO and the reported single-metal Ni-based catalysts HER catalysts in 1.0 M KOH.[1-12]

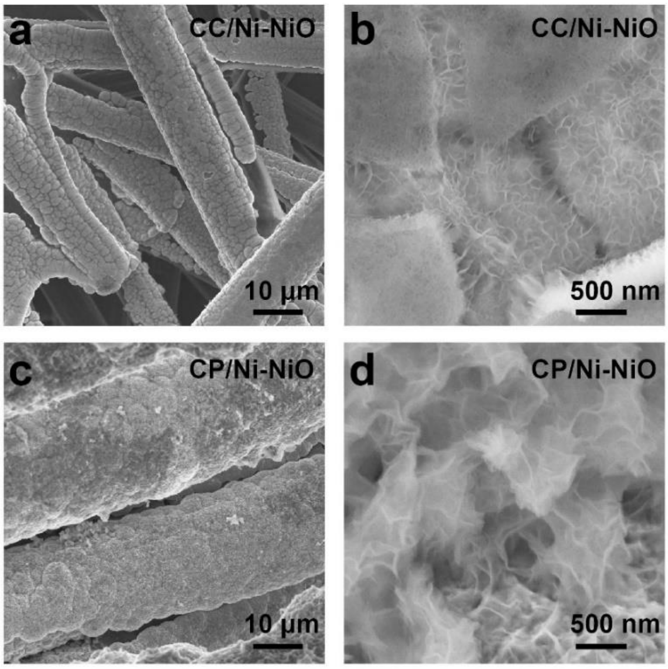

Figure S13. SEM images of Ni-NiO grown on different traditional 3D carbon electrodes. (a) CC (low magnification). (b) CC (high magnification). (c) CP (low magnification). (d) CP (high magnification)

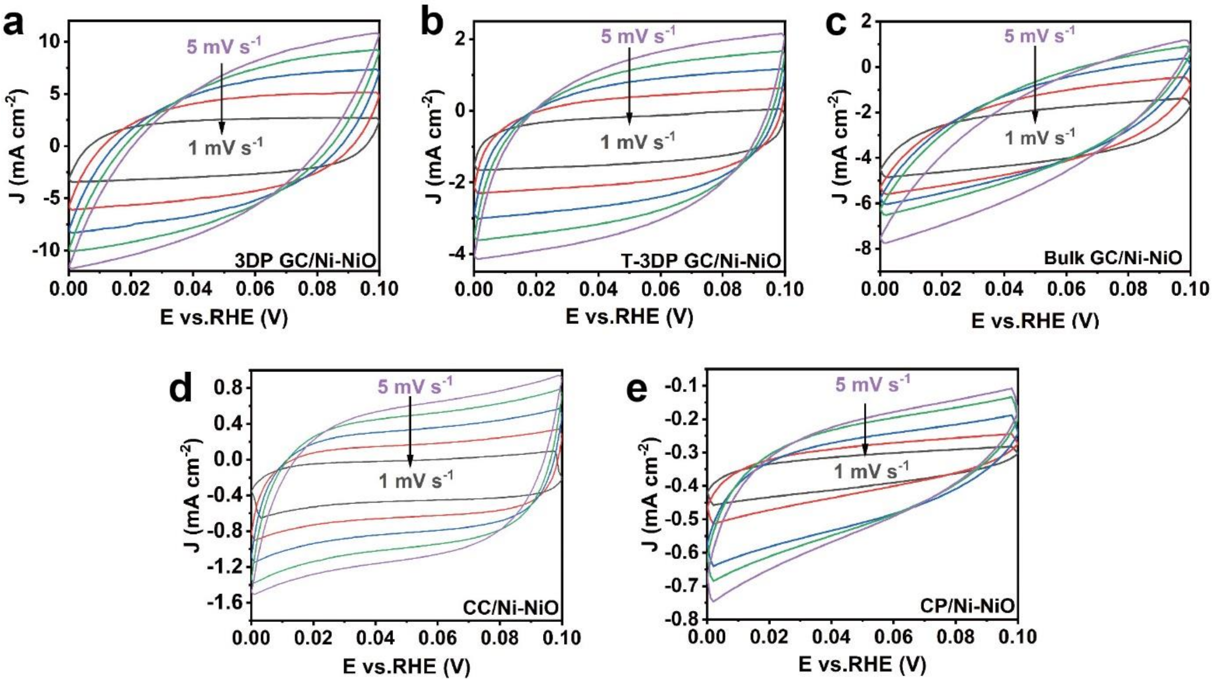

Figure S14. Cyclic voltammograms were performed in 1.0 M KOH solution in the potential window without the participation of faradaic processes: (a) 3DP GC/Ni-NiO, (b) T-3DP GC/Ni-NiO. (c) Bulk GC/Ni-NiO. (d) CC/Ni-NiO. (f) CP/Ni-NiO.

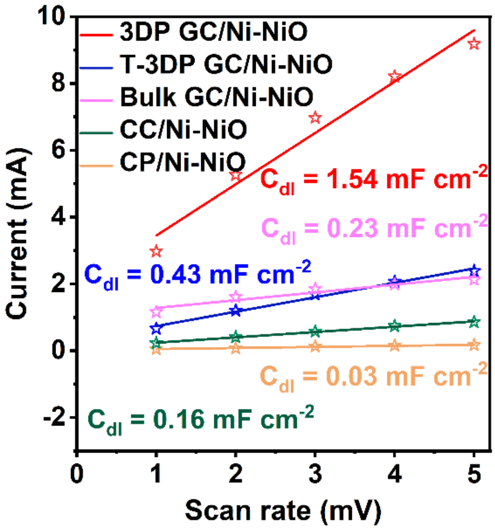

Figure S15. The double-layer capacitances (C_{dl}) of different electrodes.

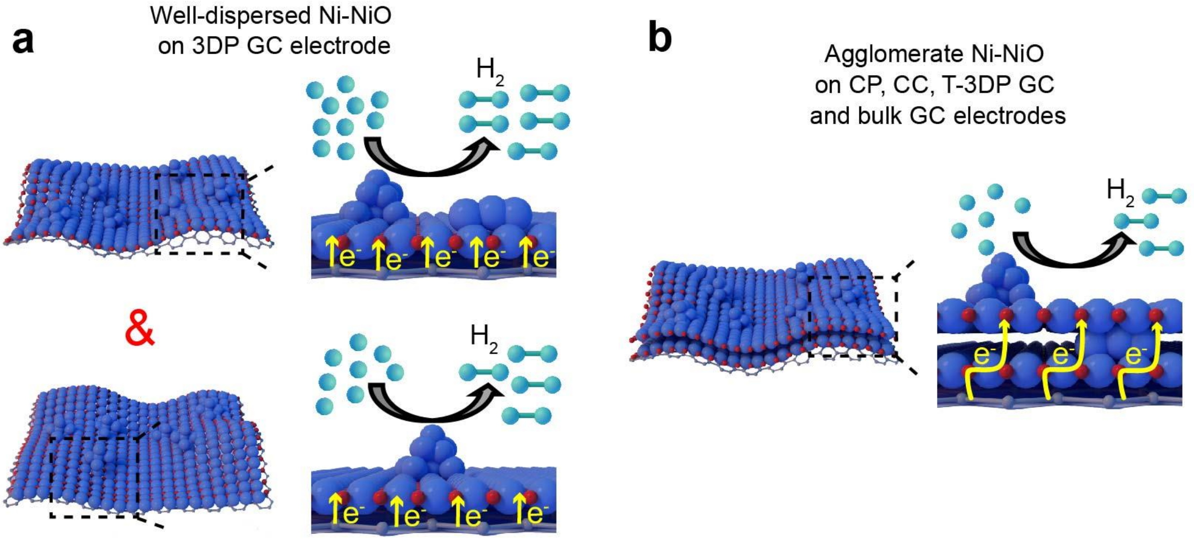

Figure S16. Schematic diagram of HER on 3DP GC/Ni-NiO, T-3DP GC/Ni-NiO, bulk GC/Ni-NiO, CP/Ni-NiO, and CC/Ni-NiO electrodes.

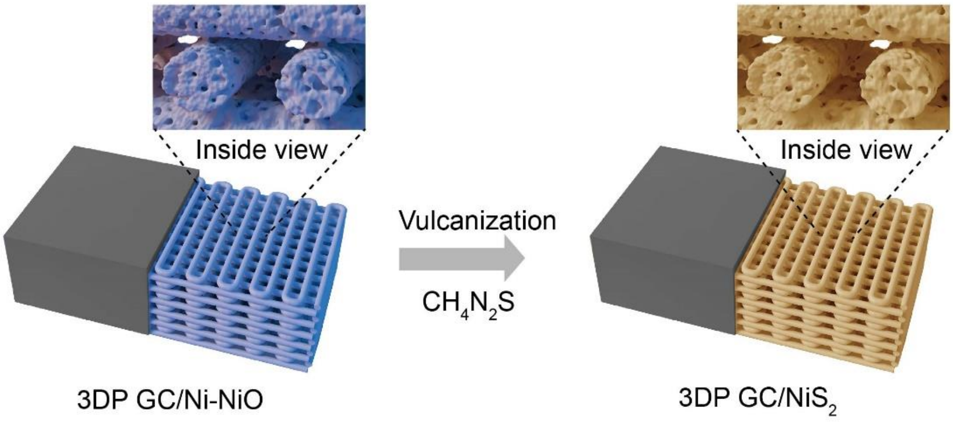

Figure S17. Schematic diagram of the 3DP GC/NiS_2 vulcanization process.

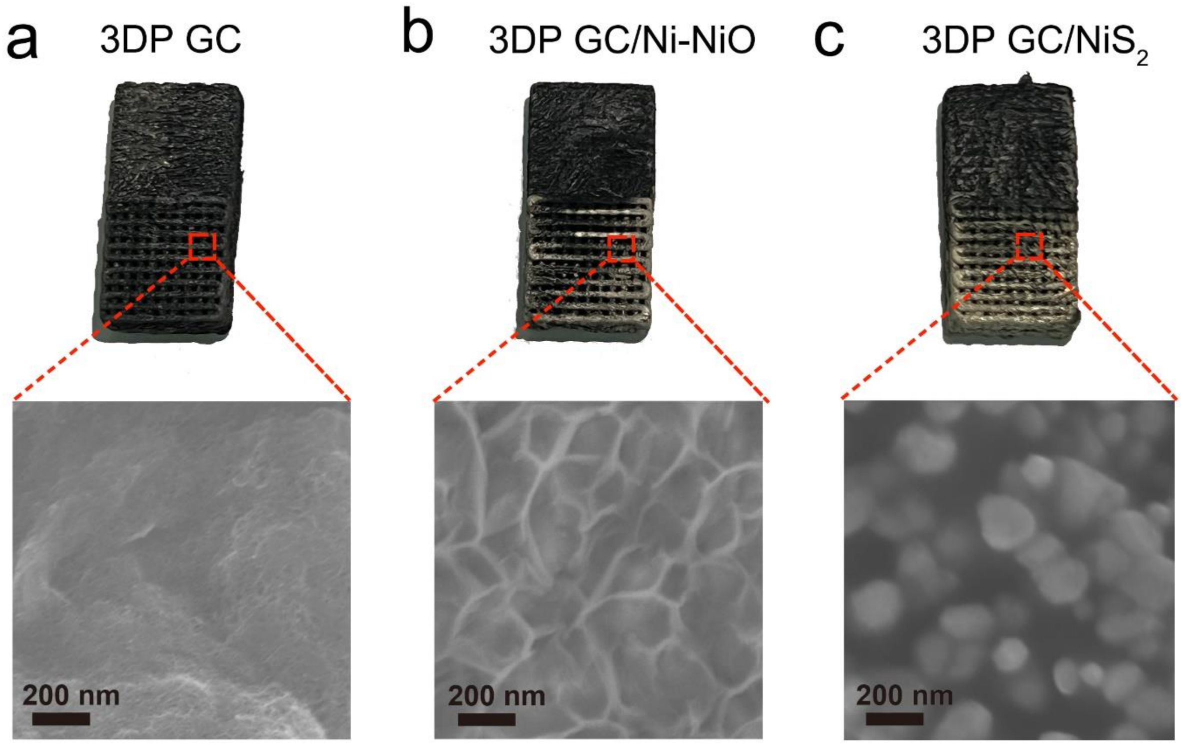

Figure S18. Optical photographs and SEM of different electrodes. a) 3DP GC b) 3DP GC/Ni-NiO c)3DP GC/NiS_2.

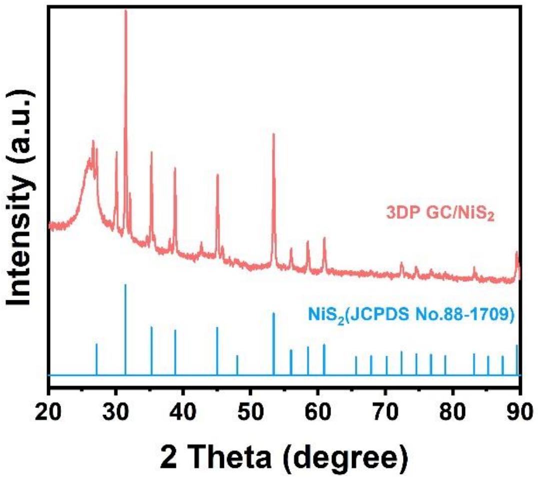

Figure S19. XRD patterns of NiS_2 on 3DP GC electrode.

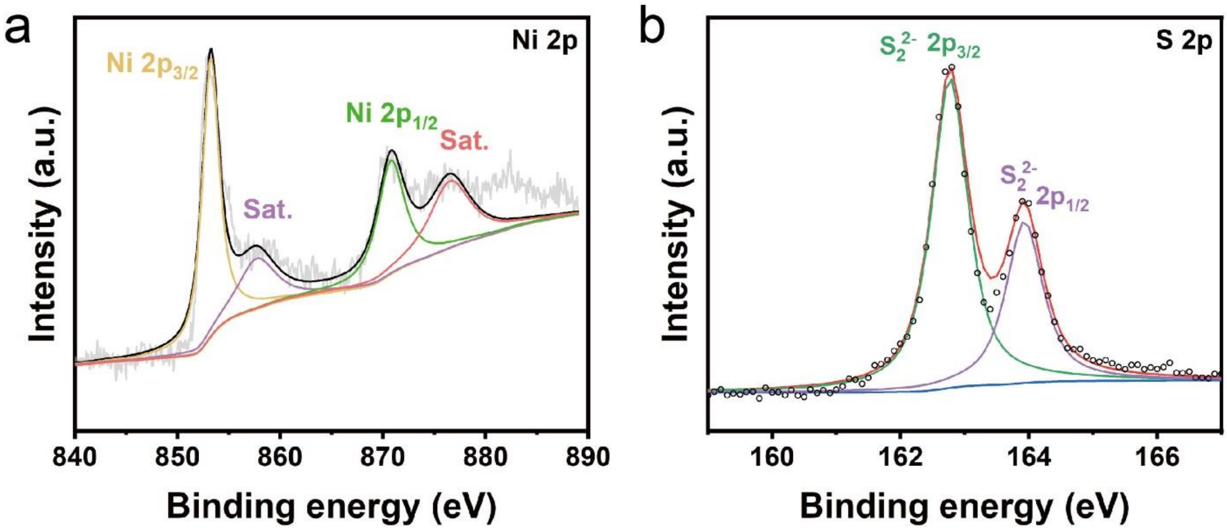

Figure S20. XPS spectra of NiS_2 grown on 3DP GC electrode. (a) Ni 2p, (b) S 2p.

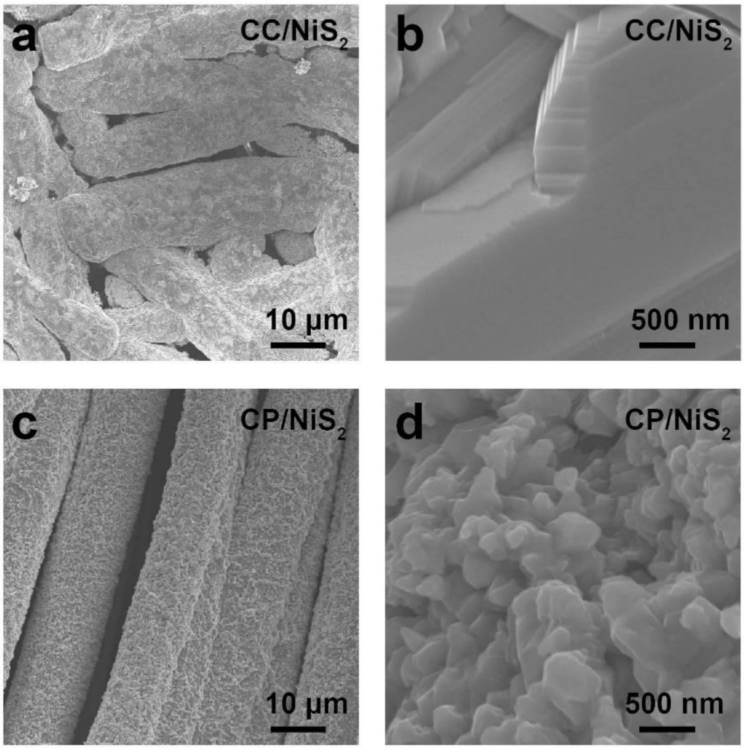

Figure S21. SEM images of NiS_2 grown on different electrodes. (a) CC/NiS_2 (low magnification). (b) CC/NiS_2 (high magnification). (c) CP/NiS_2 (low magnification). (d) CP/NiS_2 (high magnification).

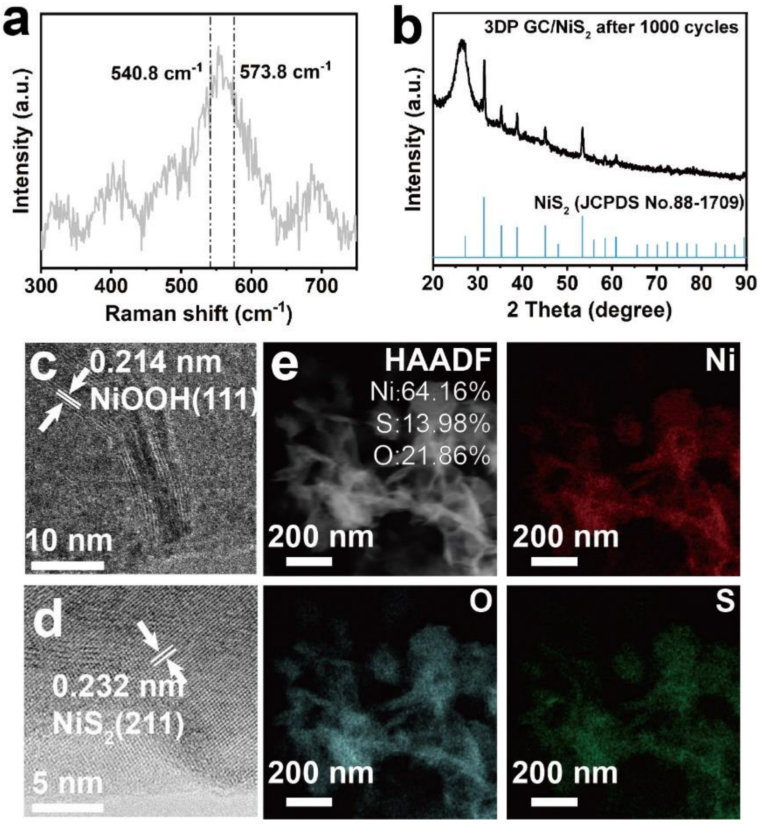

Figure S22. Characterization of the catalyst of the 3DP GC/NiS_2 electrode after the OER reaction. (a) Raman spectroscopy of NiOOH. (b) XRD pattern of NiS_2. HRTEM images of (c) NiOOH and (d) NiS_2. (e) HAADF-STEM images of the catalyst.

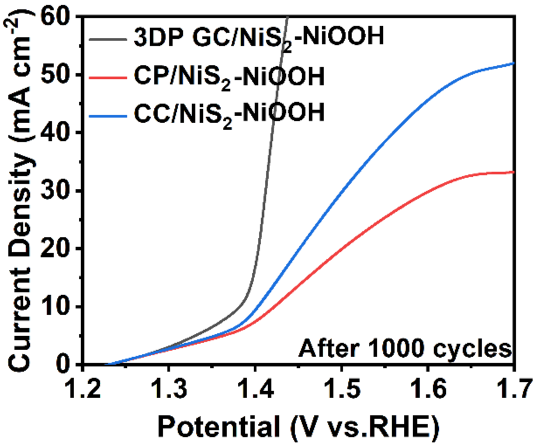

Figure S23. OER polarization curves of 3DP GC/NiS_2-NiOOH, CC/NiS_2-NiOOH and CP/NiS_2-NiOOH.

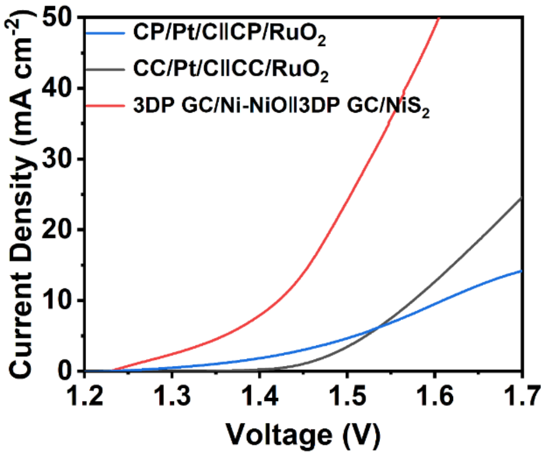

Figure S24. LSV curves of the 3DP GC/Ni-NiO||3DP GC/NiS_2, commercial CP/Pt/C||CP/RuO_2 and CC/Pt/C||CC/RuO_2 for water electrolysis in 1.0 M KOH.

Video S1. Fluid theoretical analysis of the macroscale ion transport process at the 3DP GC electrode.

Video S2. Fluid theoretical analysis of the macroscale ion transport process at bulk GC electrode.

Video S3. Fluid theoretical analysis of the microscale ion transport process at the 3DP GC electrode.

Video S4. Fluid theoretical analysis of the microscale ion transport process at the T-3DP GC electrode.

References

(1) Zang, W.; Sun, T.; Yang, T.; Xi, S.; Waqar, M.; Kou, Z.; Lyu, Z.; Feng, Y. P.; Wang, J.; Pennycook, S. J. Efficient Hydrogen Evolution Of Oxidized Ni-N_3 Defective Sites For Alkaline Freshwater and Seawater Electrolysis. *Adv. Mater.* **2021,** *33*, 2003846.

(2) Wang, L.; Fan, J.; Liu, Y.; Chen, M.; Lin, Y.; Bi, H.; Liu, B.; Shi, N.; Xu, D.; Bao, J.; Han, M. Phase-Modulation of Iron/Nickel Phosphides Nanocrystals “Armored” with Porous P-Doped Carbon and Anchored on P-Doped Graphene Nanohybrids for Enhanced Overall Water Splitting. *Adv. Funct. Mater.* **2021,** *31*, 2010912.

(3) Kou, T.; Smart, T.; Yao, B.; Chen, I.; Thota, D.; Ping, Y.; Li, Y. Theoretical and Experimental Insight into the Effect of Nitrogen Doping on Hydrogen Evolution Activity of

Ni_3S_2 in Alkaline Medium. *Adv. Energy Mater.* **2018,** *8*, 1703538.

(4) Dai, L.; Chen, Z. N.; Li, L.; Yin, P.; Liu, Z.; Zhang, H. Ultrathin Ni(0)-Embedded $Ni(OH)_2$ Heterostructured Nanosheets with Enhanced Electrochemical Overall Water Splitting. *Adv. Mater.* **2020,** *32*, 1906915.

(5) Yan, L.; Xu, Y.; Chen, P.; Zhang, S.; Jiang, H.; Yang, L.; Wang, Y.; Zhang, L.; Shen, J.; Zhao, X.; Wang, L. A Freestanding 3D Heterostructure Film Stitched by MOF-Derived Carbon Nanotube Microsphere Superstructure and Reduced Graphene Oxide Sheets: A Superior Multifunctional Electrode for Overall Water Splitting and Zn-Air Batteries. *Adv. Mater.* **2020,** *32*, 2003313.

(6) Liu, B.; He, B.; Peng, H. Q.; Zhao, Y.; Cheng, J.; Xia, J.; Shen, J.; Ng, T. W.; Meng, X.; Lee, C. S.; Zhang, W. Unconventional Nickel Nitride Enriched with Nitrogen Vacancies as a High-Efficiency Electrocatalyst for Hydrogen Evolution. *Adv. Sci.* **2018,** *5*, 1800406.

(7) Majeed, A.; Hou, P. X.; Zhang, F.; Tabassum, H.; Li, X.; Li, G. X.; Liu, C.; Cheng, H. M. A Freestanding Single-Wall Carbon Nanotube Film Decorated with N-Doped Carbon-Encapsulated Ni Nanoparticles as a Bifunctional Electrocatalyst for Overall Water Splitting. *Adv. Sci.* **2019,** *6*, 1802177.

(8) Yin, J.; Jin, J.; Zhang, H.; Lu, M.; Peng, Y.; Huang, B.; Xi, P.; Yan, C. H. Atomic Arrangement in Metal-Doped NiS_2 Boosts the Hydrogen Evolution Reaction in Alkaline Media. *Angew. Chem. Int. Ed. Engl.* **2019,** *58*, 18676-18682.

(9) Li, X.; Wang, Y.; Wang, J.; Da, Y.; Zhang, J.; Li, L.; Zhong, C.; Deng, Y.; Han, X.; Hu, W. Sequential Electrodeposition of Bifunctional Catalytically Active Structures in MoO_3 /Ni-NiO Composite Electrocatalysts for Selective Hydrogen and Oxygen Evolution. *Adv. Mater.* **2020,** *32*, 2003414.

(10) Zhou, K. L.; Wang, Z.; Han, C. B.; Ke, X.; Wang, C.; Jin, Y.; Zhang, Q.; Liu, J.; Wang, H.; Yan, H. Platinum Single-Atom Catalyst Coupled with Transition Metal/Metal Oxide Heterostructure for Accelerating Alkaline Hydrogen Evolution Reaction. *Nat. Commun.* **2021,**

12, 3783.

(11) Liu, X.; Liu, W.; Ko, M.; Park, M.; Kim, M. G.; Oh, P.; Chae, S.; Park, S.; Casimir, A.; Wu, G.; Cho, J. Metal (Ni, Co)-Metal Oxides/Graphene Nanocomposites as Multifunctional Electrocatalysts. *Adv. Funct. Mater.* **2015,** *25*, 5799-5808.

(12) Zhang, J. Y.; Liang, J.; Mei, B.; Lan, K.; Zu, L.; Zhao, T.; Ma, Y.; Chen, Y.; Lv, Z.; Yang, Y.; Yu, C.; Xu, Z.; Xia, B. Y.; Li, W.; Yuan, Q.; Zhao, D. Synthesis of Ni/NiO@MoO_{3-x} Composite Nanoarrays for High Current Density Hydrogen Evolution Reaction. *Adv. Energy Mater.* **2022,** *12*, 2200001.
